# Mice Mutated in the First Fibronectin Domain of Adhesion Molecule L1 Show Brain Malformations and Behavioral Abnormalities

**DOI:** 10.3390/biom14040468

**Published:** 2024-04-11

**Authors:** Viviana Granato, Ludovica Congiu, Igor Jakovcevski, Ralf Kleene, Benjamin Schwindenhammer, Luciana Fernandes, Sandra Freitag, Melitta Schachner, Gabriele Loers

**Affiliations:** 1 Zentrum für Molekulare Neurobiologie, Universitätsklinikum Hamburg-Eppendorf, Falkenried 94, 20251 Hamburg, Germany; v.granato@uke.de (V.G.); ludovica.congiu@u-bordeaux.fr (L.C.); ralf.kleene@zmnh.uni-hamburg.de (R.K.); sandra.freitag@zmnh.uni-hamburg.de (S.F.); 2 Institut für Anatomie und Klinische Morphologie, Universität Witten/Herdecke, 58455 Witten, Germany; igor.jakovcevski@uni-wh.de (I.J.); benjamin.schwindenhammer@uni-wh.de (B.S.); 3 Department of Neuroanatomy and Molecular Brain Research, Institute of Anatomy, Ruhr-Universität Bochum, 44780 Bochum, Germany; 4 Keck Center for Collaborative Neuroscience, Department of Cell Biology and Neuroscience, Rutgers University, Piscataway, NJ 08554, USA

**Keywords:** L1CAM, mutation, locomotion, open field, social interactions, circadian rhythm, anxiety, dentate gyrus

## Abstract

The X-chromosome-linked cell adhesion molecule L1 (L1CAM), a glycoprotein mainly expressed by neurons in the central and peripheral nervous systems, has been implicated in many neural processes, including neuronal migration and survival, neuritogenesis, synapse formation, synaptic plasticity and regeneration. L1 consists of extracellular, transmembrane and cytoplasmic domains. Proteolytic cleavage of L1’s extracellular and transmembrane domains by different proteases generates several L1 fragments with different functions. We found that myelin basic protein (MBP) cleaves L1’s extracellular domain, leading to enhanced neuritogenesis and neuronal survival in vitro. To investigate in vivo the importance of the MBP-generated 70 kDa fragment (L1-70), we generated mice with an arginine to alanine substitution at position 687 (L1/687), thereby disrupting L1’s MBP cleavage site and obliterating L1-70. Young adult L1/687 males showed normal anxiety and circadian rhythm activities but enhanced locomotion, while females showed altered social interactions. Older L1/687 males were impaired in motor coordination. Furthermore, L1/687 male and female mice had a larger hippocampus, with more neurons in the dentate gyrus and more proliferating cells in the subgranular layer, while the thickness of the corpus callosum and the size of lateral ventricles were normal. In summary, subtle mutant morphological changes result in subtle behavioral changes.

## 1. Introduction

The cell adhesion molecule L1 (L1CAM, in short L1) is a transmembrane glycoprotein of the immunoglobulin superfamily that contributes to neural functions not only during development but also in adults during synaptic plasticity and regeneration after injury [1,2]. It is evolutionarily well conserved and contains six immunoglobulin (Ig)-like and five fibronectin-type III (FNIII) domains in its extracellular part, a single transmembrane region and a cytoplasmic domain [3]. L1 plays a critical role in cell adhesion [4], axon guidance and fasciculation [5,6], neurite outgrowth [4,7,8,9,10], neuronal cell migration and differentiation [4,11,12,13], neuronal excitability [14], synapse formation and synaptic plasticity [15]. Homophilic and heterophilic interactions of full-length L1 at the plasma membrane and different L1 fragments extracellularly or intracellularly mediate different functions and lead to the activation of intracellular signaling cascades and modulate interactions with the cytoskeleton [1,2]. Plasmin [16,17], proprotein convertase PC5A [3], cathepsin E [3], MBP [3,18], presenilin/gamma-secretase [4,19,20], beta-secretase 1/BACE1 [21,22], metalloproteinases [4,23,24] and neuropsin [25,26] have been identified as L1-cleaving proteases in neural and tumor cells. The L1 fragments generated by distinct proteases are important for neurite outgrowth and neuronal survival [3,18,27,28], myelination [23], synaptic plasticity [29], mitochondrial functions [30] and regeneration after injury [31]. Several binding partners of full-length L1 or L1 fragments were identified in the extracellular matrix, at the plasma membrane, in the cytosol, in mitochondria and in the cell nucleus (see for instance: [3,27,28,29,30,32,33,34,35,36,37,38,39]).

In humans, most of the more than 300 L1 mutations presently known to localize in exons and introns cause the L1 syndrome, a rare disease with a prevalence of approximately 1/30,000, which is associated with mild to severe brain malformations, mental disabilities and spasticity [40,41,42]. Mutations were found in exons coding for the Ig-like domains, FNIII domains and cytoplasmic domain or in introns, leading to deletions, frameshifts, RNA splicing defects, abnormal localization of the protein and generation of truncated proteins (http://www.l1cammutationdatabase.info/mutations.aspx; accessed on 4 October 2023). Brain malformations, which are features of the X-chromosome-linked L1 syndrome, were also detected in mice deficient in L1 or carrying a mutation found in L1 syndrome patients [43,44,45,46,47,48,49]. Motor coordination defects were also detected in these mice [29,44]. Mental disabilities and lower expression levels of L1 were found in rats after X-irradiation at the embryonic stage [50], and L1 heterozygous female mice showed reduced social behaviors, displaying excessive self-grooming [51]. In contrast, mice ectopically expressing L1 by astrocytes showed increased behavioral flexibility and selectivity during learning and relearning [52]. These findings strengthen the importance of L1 for normal brain development and brain function. However, how mutations in L1’s proteolytic cleavage sites and the absence of L1 fragments affect L1’s functionality during brain development and in the adult brain is not well understood. To investigate how a mutation in the MBP cleavage site in the first FNIII domain of L1 affects brain development, function and, on the molecular side, L1-mediated cellular and biochemical processes, we generated gene-edited mice expressing L1 with the exchange of arginine 687 to alanine (R/A687 substitution; L1/687 mice) (Supplementary Figure S1). The R to A substitution at position 687 disrupts the MBP cleavage site R687V in L1 and causes the absence of a 70 kDa L1 fragment [53], which is imported into mitochondria and cell nuclei and is essential for L1’s functions in vitro [18,27,29,30,54]. Importantly, the level of full-length L1 in the brains of L1/687 mice was similar to the L1 level in wild-type mice [27,53], and basal neurite outgrowth, neuronal survival and Schwann cell process formation were comparable to the outgrowth and survival of wild-type cells, while neuronal migration was reduced [54]. In addition, L1/687 neurons were impaired in outgrowth, migration and survival when L1 was stimulated and did not respond to the triggering of L1 through the addition of L1 agonists or antibodies [54]. Mitochondrial functions were also impaired in L1/687 neurons, and mitochondria moved more retrogradely [54]. Furthermore, we showed that L1-70 interacts with microtubule-associated protein 1A/1B-light chain 3, topoisomerase I, peroxisome proliferator-activated receptor γ and β-nicotinamide adenine dinucleotide hydrate dehydrogenase (ubiquinone) flavoprotein 2, and, in L1/687 cells, these interactions did not take place. In contrast, the retinoid X receptor, estrogen receptor α, splicing factor proline/glutamine-rich, non-POU domain-containing octamer-binding protein and paraspeckle component 1 interacted with L1 with R687V mutation and did not bind to L1-70 [27,55]. We analyzed in vivo the influence of the R to A substitution at position 687 of L1 on brain development and nervous system functions in adult mice. We observed enhanced locomotion and changes in social interactions and, at more advanced ages, reduced motor coordination. Anxiety levels and circadian rhythm were not altered in the mutant. Histological and immunohistochemical analyses showed normal thickness of the corpus callosum and lateral ventricles of L1/687 males and females, whereas L1/687 males and females had a larger hippocampus than their wild-type littermates. In addition, the hippocampi of L1/687 mice exhibited more granule cells in the dentate gyrus and a higher number of proliferating neural stem cells in the subgranular layer. Altogether, the combined results indicate that the mutation of MBP’s cleavage site in L1’s first FNIII domain does not result in gross anatomical abnormalities but in some sex- and age-dependent phenotypes, which remain to be understood in terms of molecular mechanisms.

## 2. Materials and Methods

### 2.1. Mice

Gene-edited mice expressing L1 with an R to A substitution at position 687 in the first FNIII domain (L1/687 mutant mice) have been described [53]. Mice were bred using heterozygous females and wild-type (WT) or L1/687 males. Mating of WT males and heterozygous females yields WT and L1/687 males as well as heterozygous and WT females. Mating of L1/687 males with heterozygous females yields WT and L1/687 males and heterozygous and L1/687 females. Mice were maintained at the Universitätsklinikum Hamburg-Eppendorf on a 12 h light/12 h dark cycle under standard housing conditions (21 ± 1 °C, 40–50% humidity, food and water ad libitum). Three- to five-month-old L1/687 males and their male WT littermates were used for behavioral studies. Three- to five-month-old L1/687 females and female WT cousins were used for behavioral studies, since L1/687 females and WT females cannot be obtained from the same parents (see above for mating results). We also used eight-month-old mice of both genotypes for the Rotarod test and for morphological analysis. Experiments were approved by the Behörde für Justiz und Verbraucherschutz of the State of Hamburg (animal permit number N 073/2020 (approval date 16 September 2020)). Experiments were designed, and the manuscript was prepared according the ARRIVE guidelines [56].

### 2.2. Reagents

Reagents were purchased from Sigma-Aldrich (Taufkirchen, Germany) or Carl Roth (Karlsruhe, Germany) unless otherwise indicated.

### 2.3. Histology and Immunohistochemistry

One day after the last behavioral tests, animals were deeply anesthetized by intraperitoneal injections of ketamine (CAS 6740-88-1) and xylazine (CAS 7361-61-7) (80 mg Ketanest^®^ (Pfizer Pharma PFE GmbH, Berlin, Germany) and 10 mg Xylazine^®^ (WDT, Garbsen, Germany), per kg body weight) and then transcardially perfused with fixative (4% *w*/*v* formaldehyde (CAS 50-00-0) and 0.1% *w*/*v* CaCl_2_ in phosphate-buffered saline (PBS), pH 7.4). Brains were isolated, postfixed for at least 24 h at 4 °C in the same fixative and then immersed in a 20% *w*/*v* sucrose solution in PBS, pH 7.4, for 2 days at 4 °C, followed by immersion in 30% *w*/*v* sucrose solution in PBS, pH 7.4, for 2 days at 4 °C. Brains were frozen by a 2 min immersion in 2-methylbutane (CAS 78-78-4), precooled to −80 °C and stored at −80 °C until use. Brains were cut into 25 µm thick coronal sections on a cryostat (Leica CM3050, Leica Instruments, Nußloch, Germany), collected on SuperFrost Plus glass slides (Carl Roth) and stored at −20 °C until use. Sampling of sections was always performed in a standard sequence so that four sections, 250 μm apart, were present on each slide.

For immunofluorescence staining, cryosections were left at room temperature (RT) to dry for 1 h. After a brief wash in PBS, the sections were incubated in blocking solution containing 5% *w*/*v* normal goat serum and 0.2% Triton X-100 (CAS 9036-19-5) dissolved in PBS for 1 h at RT to reduce non-specific binding. Sections were then incubated overnight at 4 °C with its primary antibody, diluted in blocking solution. The following commercially available primary antibodies were used for immunofluorescence staining: anti-neuron specific nuclear antigen (NeuN; RRID:AB_2298772; mouse monoclonal, clone A60, 1:1,000; Merck, Darmstadt, Germany), anti-glial fibrillary acidic protein (GFAP) (RRID:AB_10013382; 1:500; DakoCytomation, Hamburg, Germany, rabbit polyclonal), anti-allograft inflammatory factor 1 (Iba1) (RRID:AB_839504; rabbit polyclonal, 1:1,500; Wako Chemicals, Neuss, Germany) and anti-Ki67 (RRID:AB_302459; 1:500, rabbit, polyclonal, Abcam). After washing in PBS (3 × 10 min at RT), the appropriate Cy2- or Cy3-conjugated secondary antibody (Jackson ImmunoResearch, Cambridgeshire, United Kingdom) diluted 1:200 in PBS was applied for 2 h at RT. After subsequent washes in PBS (3 × 10 min at RT), the sections were mounted in anti-quenching medium, with the addition of 4′,6-diamidino-2-phenylindole (DAPI; CAS 108-88-3), which visualizes cell nuclei (RotiMount with DAPI; Carl Roth), and stored in the dark at 4 °C.

Hematoxylin (CAS 517-28-2) and eosin (CAS 517-28-2) (HE) staining was performed using a standard protocol. Briefly, the sections were rehydrated in xylene and ethanol (2 times 100% xylene (CAS 1330-20-7) followed by 100% ethanol, 100% ethanol, 95% ethanol and 75% ethanol, each 5 min) and stained with hematoxylin–eosin (hematoxylin 2 min, eosin 30 s). Afterwards, the sections were dehydrated in ethanol and xylene and sealed with Entellan (CAS 108-88-3; Sigma-Aldrich).

### 2.4. Quantitative Morphological Analysis

Volumes of the lateral ventricles, hippocampus and hippocampal subfields were estimated according to Cavalieri’s principle, using area measurements obtained on an Axioskop microscope (Zeiss, Oberkochen, Germany) equipped with a motorized stage and Neurolucida software-controlled computer system (MicroBrightField Europe, Magdeburg, Germany). The same microscope and software were used to measure average thickness of hippocampal principal cell layers and corpus callosum. To estimate cell densities (number of cells per volume), the optical dissector method was used, as described [57]. The counts were performed under an Axioskop microscope (Zeiss) equipped with a motorized stage and Neurolucida software-controlled computer system (MicroBrightField Europe). Cell densities were estimated in every 10th spaced serial section (250 μm apart) in which the areas of interest were seen. Counting was based on the identification of the position of the cell nuclei of immunostained cells within the dissector using a 40× objective. The parameters for the stereological analysis were as follows: guard space depth 2 μm, base and height of the dissector 3600 μm^2^ and 10 μm, respectively, distance between the optical dissectors 60 μm and objective of Plan-Neofluar^®^ 40×/0.75 (Zeiss). The same parameters were used for counting the nuclei in the pyramidal and granular cell layers, except for the base of the dissector and in the space between dissectors, which were 625 μm^2^ and 25 μm, respectively. Left and right cortical and hippocampal areas were evaluated. All results shown are averaged bilateral values. Since Ki67-positive cells in the subgranular layer of the dentate gyrus (DG) were rare, they were counted for the total DG.

### 2.5. Behavior

Mice were housed in groups of three to six mice (maximally two mice from one litter per group) and accustomed to an inverted day–night cycle (light off at 7:00 am and light on at 7:00 pm) for one week and then accustomed to the experimenter by handling for one hour per day for one week. Before the experiments, mice were transported to the experimental room next to the vivarium, which was illuminated by dim red light, and left for 5–10 min for habituation. Tests started and ended at least 2  h after light offset and 3  h before light onset. After each test, the mazes were cleaned with soap and water and then with 30% ethanol. Care was taken to minimize pain or discomfort for the animals. Tracks representing the position of the mice were created and analyzed with EthoVision (Noldus, Wageningen, The Netherlands; https://www.noldus.com/ethovision; RRID:SCR_000441; accessed 5 June 2021) [58,59]. Manual scoring of behavior was performed by a trained experimenter blinded to the genotype of the mice using The Observer software (Noldus). Randomization of mice was performed according to the ARRIVE guidelines. Numbers of mice used per group are indicated in the figure legends.

A timeline of behavioral experiments is shown in Supplementary Figure S2.

#### 2.5.1. Open Field and Elevated Plus Maze

To evaluate activity levels and exploratory and anxiety-like behavior, mice were evaluated in the open filed and elevated plus maze [59,60]. A multiple unit open field (OF) consisting of four activity chambers was used. Each OF chamber of 50 cm (length) × 50 cm (width) × 50 cm (height) was made from white high-density and non-porous plastic and was illuminated with 50 lux. Then, a mouse was placed in an acrylic glass cylinder located in one corner of each activity chamber. When the cylinder was lifted, the mice could freely move in the arena for 20 min, and the distance moved and behavior in the arena were recorded. In addition to the time spent in the center of the arena, the total distance moved and the average distance from the wall, rearing on and off the wall, self-grooming time and immobility time were analyzed.

The elevated plus maze consisted of a plus-shaped arena with four arms, 30 cm long and 5 cm wide, connected with a center zone of 5 × 5 cm. Two opposing arms were bordered with a 2 mm rim (open arm), while the other two had a 15 cm high wall (closed arm). The maze was elevated 75 cm above the floor and illuminated with 10 lux. Mice were placed in the center of the maze facing the open arm and observed for 5 min. The following parameters were analyzed: open and closed arm entries, calculated when all the four paws were inside the arm, and stretch attend posture, calculated when the mouse stretched forward and retracted to the original position without forward locomotion, as well as self-grooming and head dips.

#### 2.5.2. Social Interaction

Motivation to investigate a social stimulus was tested by allowing the mouse to choose between attending to an unfamiliar sex-matched mouse or a familiar mouse [58]. The arena used for the open field test (50 × 50 cm) was illuminated with 5 lux and divided into two identical compartments by a 40 cm high wall with an opening in the middle, allowing the mouse access to both compartments. A cylinder with a metal grid mesh allowing for olfactory exploration between mice was located in one corner of each compartment, containing either a familiar or an unfamiliar mouse. Familiar mice were recruited from heterozygous or WT siblings from the same cage as the experimental mice. Unfamiliar mice were heterozygous or WT mice that were from other cages and not used as subjects in the behavior tests. First, the familiar and unfamiliar mice were placed under the cylinders. The subject mouse was then placed in the arena and left free to move between compartments for 20 min. The room was illuminated with 5 lux during recordings, and distance moved, time spent in each compartment and time spent in proximity to the two cylinders were determined. The time spent exploring the unfamiliar mouse was divided by the time exploring the familiar and unfamiliar mouse. Values higher than 0.5 indicate a preference to explore the unfamiliar mouse.

#### 2.5.3. Rotarod, Beam Walking and Pole Test

To evaluate motor coordination, the Rotarod test, beam walking test and pole test were used. For the Rotarod test [61,62], mice were trained on a rotating rod with 3 cm diameter (Rotarod for mice, UGO BASILE S.R.L., Schwerte, Germany) for two days with two trials with constant speed (4 rpm) for 2 min and three trials of training with acceleration (4–40 rpm) for 4 min. After every training trial, the training was stopped for an interval of 10 min before continuing with the next training phase. For the next three to four consecutive days, mice were subjected to the test; mice were located on the 3 cm diameter rod, with acceleration from 4 to 40 rpm in 5 min. The tests were performed under red light, and the latency to fall from the rod was recorded. In the beam walking test, mice were trained to cross a wooden beam (90 cm long and 5 cm wide, positioned 50 cm above the ground) in order to reach their home cage at the end of the beam. To further motivate mice, food was placed at the end of the beam. During the trial, mice were recorded from behind while walking on the beam towards their home cage, from the beam end positioned with the experimenter, and the time to cross the bream, the heels–tail angle and the foot–base angle were determined [63]. Experiments were performed at 50 lux. In the pole test, motor coordination was monitored while mice were climbing down a pole. The test was performed as described [58]. Mice were placed head upward at the top end of a rough-surfaced vertical wooden rod (48.5 cm long, 3 mm diameter). The time needed by the mice to reach the floor with all four paws and their ability to turn 180° were analyzed.

#### 2.5.4. Circadian Activity

Mice were housed singly in a type II cage with a size of 23 × 20 × 15 cm for 3 days, and then the circadian activity during a 24 h day–night cycle was recorded with a motion detector (Mouse-E-Motion; Infa-E-Motion, Hamburg, Germany) that was placed on top of the cage. The activity of the mice was recorded in 4 min time bins, and E-motion software was used to analyze the activity of the mice [58]. 

#### 2.5.5. Marble Burying

Digging behavior and activity were evaluated in the marble burying test [64,65]. The test was performed under red light and in a 42 × 24 × 12 cm cage. A 5 cm layer of fresh bedding material covered the floor of the cage, and 20 black marbles (diameter 1.5 cm) were placed on top of the bedding. The mice were placed in one corner of the cage and left for 30 min to explore and move before being returned to the home cage. Numbers of buried marbles were counted and scored.

### 2.6. Statistics

All numerical data are presented as single values and group mean values with standard error of the mean (SEM) or as group mean values with standard deviation (SD). Normal distribution of the data was determined using Shapiro–Wilk test and Levene’s test before choosing the appropriate statistical test. Statistical tests used for comparisons are indicated in the figure legends. Analyses were performed using SPSS or GraphPad software.

## 3. Results

### 3.1. Behavioral Analysis

#### 3.1.1. Body Weight, Muscle Function and Motor Coordination

Mutations of L1 in humans often lead to impaired motor abilities, as seen in the varying spasticity of lower limbs in most patients [41,66,67,68]. We, therefore, examined motor coordination in the L1/687 mutants. To preclude that muscle weakness or changes in body weight underlie defects in motor coordination, we first analyzed body weight and quadriceps muscle function. Body weight was determined for male and female L1/687 mice and WT mice at the age of three weeks up to five months. Average weight ± SD at five months for WT males was 33.05 ± 3.49 g and for L1/687 males it was 30.49 ± 3.12 g (*p* = 0.098, *n* = 10 mice per group); thus, male L1/687 mice tended to be insignificantly smaller and lighter compared to their WT littermates. Female L1/687 mice were significantly lighter compared to the WT females at five months. WT females had an average weight ± SD of 23.77 ± 2.24 g and L1/687 females had a weight of 21.77 ± 1.63 g (*p* = 0.042, *n* = 9 mice per group; unpaired *t*-test). When evaluating quadriceps muscle function in the beam walking test, L1/687 mice spent a similar amount of time to cross the beam, and plantar stepping abilities, as determined by foot–base angle and heels–tail angle, were not different compared to their WT littermates (Supplementary Figure S3).

**Figure 1:**
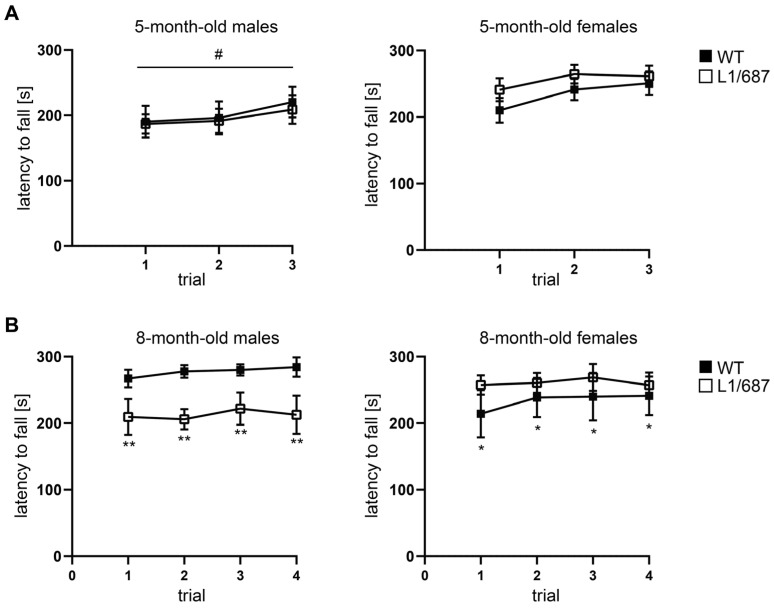
Motor performance of L1/687 mice in the Rotarod test. Five-month-old (**A**) and eight-month-old (**B**) WT and L1/687 males and females were subjected to the Rotarod test and the latency to fall from the accelerating rod during three to four consecutive trials was determined. Average values ± SEM are shown; *n* = 10 males and 9 females (5 months old), *n* = 8 males and 5 females (8 months old). Two-way ANOVA and Tukey’s multiple comparison post hoc test, * *p* < 0.05, ** *p* < 0.01 (comparison between genotypes), # *p* < 0.05 (comparison between first and last trial).

To assess motor coordination, the pole test and the Rotarod test were performed. All mice were able to turn 180° to climb down the vertical pole, and the time needed to climb down was similar for all genotypes (Supplementary Figure S4). In addition, all mice performed the task more quickly in the second trial. On the accelerating Rotarod, the latency to fall from the rod was similar for five-month-old male and female L1/687 mice when compared to WT males and females (Figure 1A). Since motor performance declines with age [69,70], aging might affect the phenotype of the L1/687 mutant mice differently compared to WT mice, so we also used older mice to study their coordination. Eight-month-old female L1/687 mice had a higher latency to fall from the accelerating rod than WT females, while male L1/687 mice fell off the rod sooner than their WT male littermates (Figure 1B). Interestingly, motor coordination was different between genders in 8-month-old mice, and L1/687 females performed better than L1/687 males (Figure 1B).

#### 3.1.2. Exploratory Behavior in the Open Field and Elevated Plus Maze

**Figure 2:**
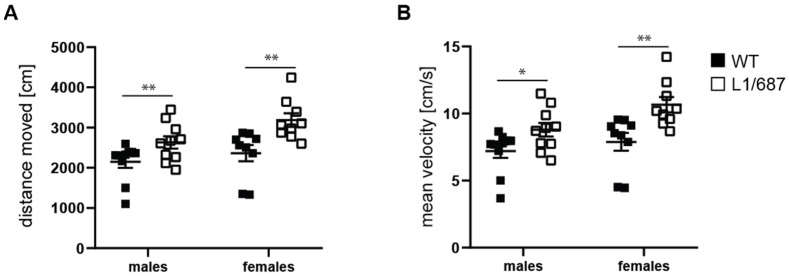
Higher activity of L1/687 mice in the open field test during the first minutes. Three-month-old WT and L1/687 males and females were subjected to this test, and distance moved (**A**) and velocity (**B**) were determined during the first 5 min. Average values ± SEM are shown; *n* = 9–10 mice per group; two-way ANOVA and Bonferroni post hoc test, * *p* < 0.05, ** *p* < 0.01.

**Figure 3:**
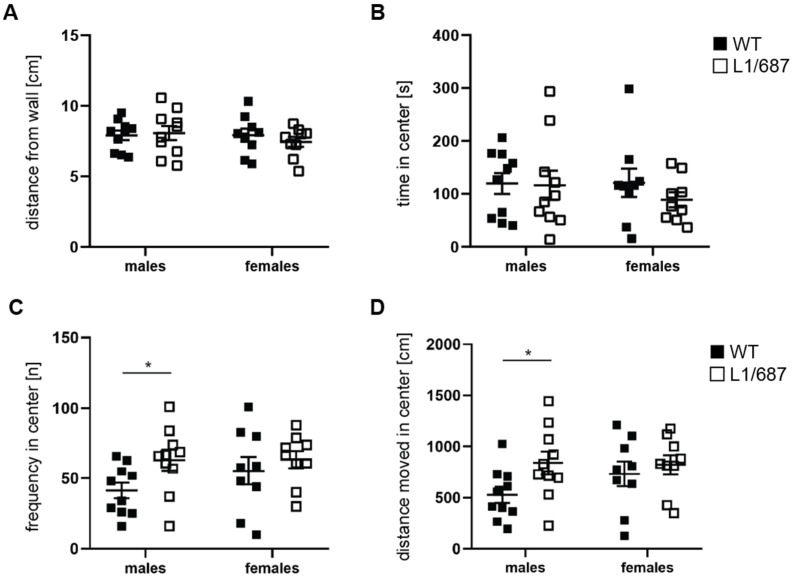
Male L1/687 mice enter the center of the open field at a higher frequency and move a longer distance in the center. Three-month-old WT and L1/687 males and females were evaluated over 20 min for mean distance to the wall (**A**), time in the center zone (**B**), frequency to enter the center zone (**C**) and distance moved in the center zone (**D**). Single and average values ± SEM are shown; *n* = 9–10 mice per group; two-way (distance from the wall and time in the center) and one-way ANOVA (frequency and distance moved in the center) and Bonferroni post hoc test, * *p* < 0.05.

Reduced social functioning, depression and reduced self-confidence are features of L1 syndrome patients [41,67,71,72]. Therefore, we evaluated exploratory behavior and anxiety in the open field and elevated plus maze. In the open field, male and female L1/687 mice moved faster and over a longer distance in the first five to ten min of the test compared to WT mice (Supplementary Figure S5 and Figure 2), while there were no differences between genotypes after habituation (Supplementary Figure S5). Distance to the wall and time in the center zone (Figure 3), self-grooming time, rearing number and immobility time (Supplementary Figure S6), which are characteristic of the anxiety state, were similar for the genotypes. Interestingly, L1/687 males entered the center zone of the open field more often and moved a longer distance in the center compared to WT males, while L1/687 females did not differ from WT females (Figure 3). The results suggest that the locomotion of L1/687 males and females is enhanced. Moreover, overall anxiety-related parameters of L1/687 females are comparable to those of WT females, while L1/687 males are either less anxious or more active in the center of the arena than WT males.

**Figure 4:**
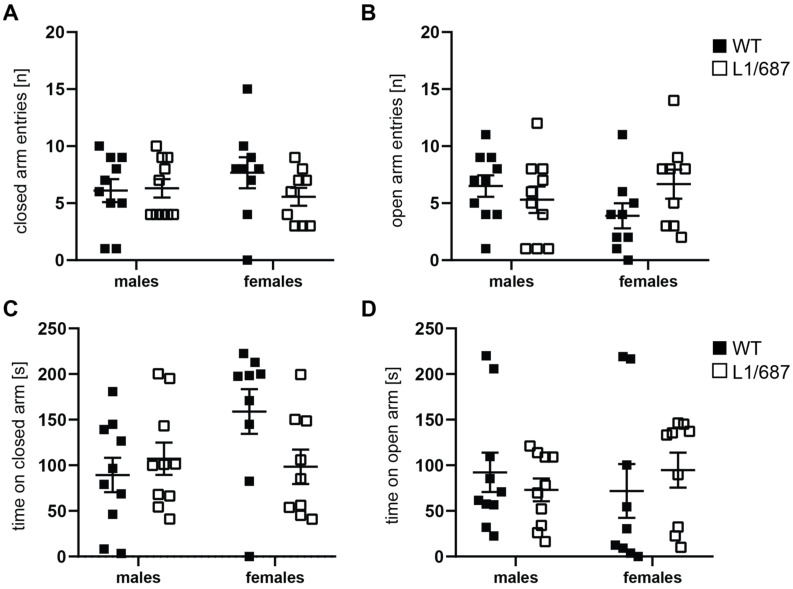
Normal anxiety of L1/687 mice in the elevated plus maze. Three-month-old WT and L1/687 males and females were subjected to the elevated plus maze, and closed and open arm entries (**A**,**B**) and times spent in the closed and open arms (**C**,**D**) were determined during 5 min. Single and average values ± SEM are shown; *n* = 9–10 mice per group; two-way ANOVA and Bonferroni post hoc test; no significant differences were found, *p* > 0.05.

The exploratory behavior was additionally examined in the elevated plus maze. L1/687 males and females were not different from WT males and females and spent comparable amounts of time in the open and closed arms of the maze (Figure 4A,B). WT males stayed 31 ± 22.7% of the time in the open arms, 30 ± 19.7% in the closed arms and 39 ± 27.3% in the center; L1/687 males stayed 24 ± 13.1% of the time in the open arms, 36 ± 18.6% in the closed arms and 40 ± 29.3% in the center; WT females spent 24 ± 29.5% of the time in the open arms, 53 ± 24.5% in the closed arms and 23 ± 11% in the center; L1/687 females stayed 32 ± 19.2% of the time in the open arms, 33 ± 18.8% in the closed arms and 35 ± 18.6% in the center. Compared to WT males, L1/687 males showed a reduced immobility time, while females of both genotypes were not different from each other (Supplementary Figure S7). As seen for to the open field, no noteworthy differences in anxiety-related parameters were observed between genotypes. Regarding the open field and elevated plus maze, we can conclude that L1/687 males and females exhibit an enhanced locomotor activity but no changes in anxiety-related parameters.

#### 3.1.3. Social Interaction

**Figure 5:**
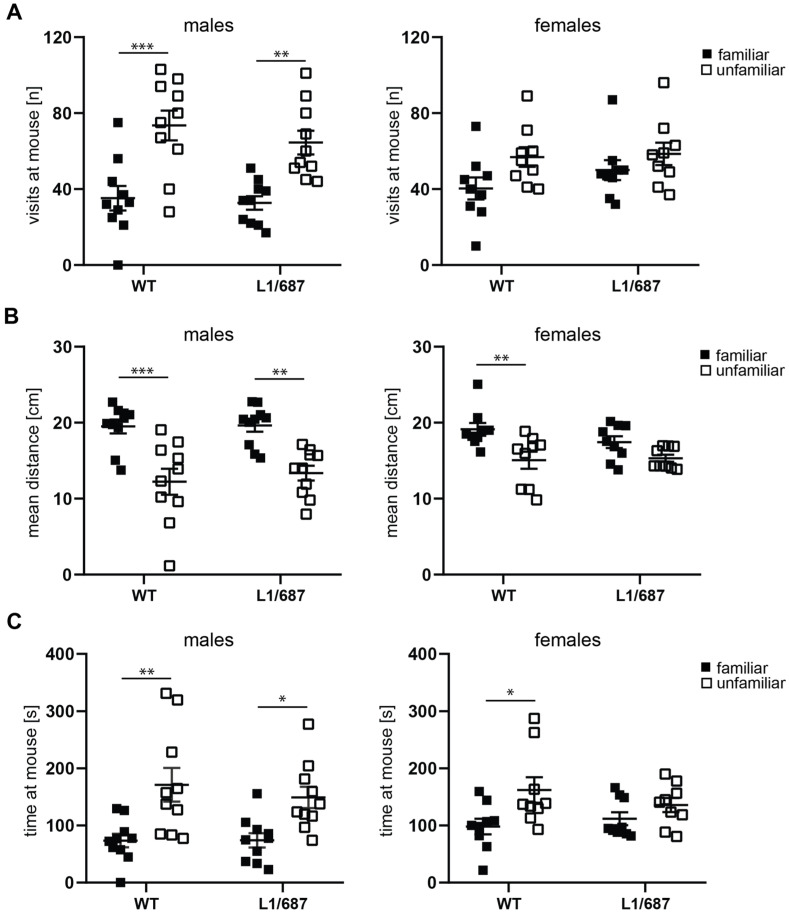
Impaired social interest of L1/687 females. Four-month-old WT and L1/687 males and females were subjected to the social interaction test, and visits to the familiar and unfamiliar mouse (**A**), distance to the familiar and unfamiliar mouse (**B**), and time spent with the familiar and unfamiliar mouse (**C**) were recorded during 10 min. Single and average values ± SEM are shown; *n* = 9–10 mice per group; two-way ANOVA and Tukey’s multiple comparison post hoc test, * *p* < 0.05, ** *p* < 0.01, *** *p* < 0.001.

To assess social novelty interest and social memory and to determine if L1/687 mice present autism-related behavioral deficits like L1 heterozygous female mice, and since L1 is associated with Rett syndrome and autism [51,73,74], social interaction was measured. Mice were exposed to a familiar and unfamiliar mouse presented in a cage with mesh wire to allow for olfactory and visual contact. The number of visits and time spent near to the unfamiliar and familiar mouse and the distance moved by the subject mouse close to the conspecific stranger in a wire cage, versus the chamber containing the known mouse from the same home cage, were measured, and the preference index was calculated. Males of both genotypes visited the unfamiliar mouse more often than the familiar mouse, while females only visited the unfamiliar mouse slightly more than the familiar mouse (Figure 5A and Supplementary Figure S8). Males of both genotypes and WT females stayed at a larger distance from the familiar mouse and spent a longer time close to the unfamiliar mouse compared to the familiar mouse, while L1/687 females did not distinguish between the familiar and unfamiliar mouse or were more anxious to approach the unfamiliar mouse and stayed the same time and at the same distance to the familiar and unfamiliar mouse (Figure 5B,C). The results show that L1/687 females are impaired in social interest and novelty seeking, while L1/687 males are not different from their WT littermates.

#### 3.1.4. Marble Burying Test

**Figure 6:**
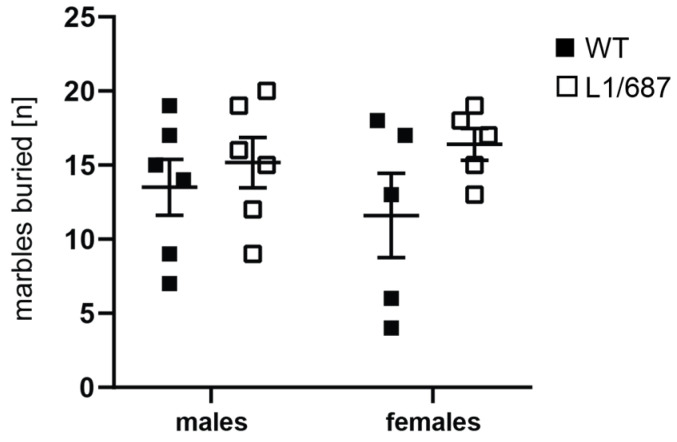
L1/687 mice show normal behavior in the marble burying test during 30 min. Numbers of marbles buried were counted for five-month-old WT and L1/687 males and females. Single and average values ± SEM are shown, *n* = 5–6 mice per group; two-way ANOVA and Tukey’s multiple comparison post hoc test; no significant differences were found, *p* > 0.05.

Since obsessive–compulsive behavior and altered reaction to novelty are common in patients with autism spectrum disorders, and since L1 syndrome can also be associated with autistic-like behavior in humans and mice [33,51,72,73,74,75,76], we examined L1/687 mice in the marble burying test to evaluate digging activity, to observe repetitive and compulsive-like behaviors or changes in anxiety, activity and novel object recognition. Mice were placed in a cage containing high bedding with 20 black marbles on top. Mice were allowed to explore the cage, and the numbers of marbles buried were determined. No differences between L1/687 and WT mice were observed (Figure 6). The results show that L1/687 mice do not exhibit obsessive–compulsive behavior or differences in reaction to novel objects compared to WT mice.

#### 3.1.5. Circadian Activity

The circadian rhythm and activity levels of single-housed mice were examined in their home cage for 24 h. All mice were more active during the dark phase compared to the light phase (Supplementary Figure S9). L1/687 males and females were not different in activity in the dark and light phases when compared to their WT littermates. Of note in this context, L1/687 mice were more active in the open field, which could be a reaction to the novel environment. L1/687 males and females were normal in terms of circadian rhythm and in spontaneous behavior in their home cage, indicating that behavior in a familiar environment is similar to that of WT mice.

#### 3.1.6. Brain Structure

**Figure 7:**
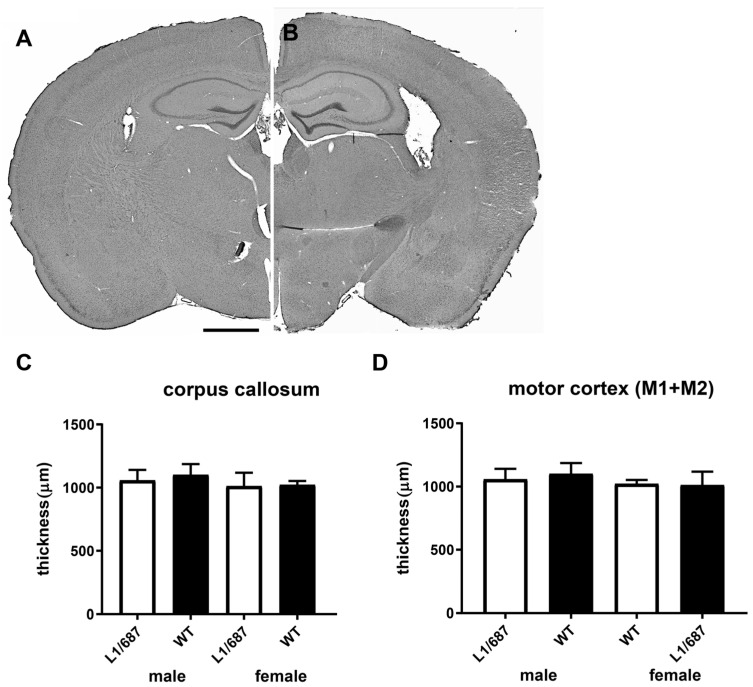
Normal thickness of the corpus callosum and motor cortex of L1/687 mice. Shown is a representative HE stained coronal brain section from a female L1/687 (**A**) and a female WT (**B**) mouse. Scale bar: 1 mm. Average thickness of corpus callosum (**C**) and motor cortex (**D**) in L1/687 and WT mice. Shown are mean values + SD, *n* = 7 mice per group; *t*-test; no significant difference, *p* > 0.05.

Changes in brain anatomy and function could underlie the changes seen in L1/687 mouse behavior. In patients with L1 syndrome as well as in L1-deficient mice and in L1 syndrome model mice slightly to severely enlarged ventricles, and changes in the thickness of the corpus callosum were found [42,43,44,45,49,66,68]. These alterations can lead to further changes in brain structure, resulting in changes in neural functions. Therefore, we next investigated if L1/687 mice display abnormalities in brain structure. We first measured the lateral ventricle size from the hematoxylin/eosin (HE)-stained sections in five-month-old L1/687 and WT littermates (Figure 7A,B). The ventricle volumes were similar between L1/687 and WT mice (0.95 ± 0.21 µm^3^ vs. 0.88 ± 0.34 µm^3^, respectively; *p* > 0.05, Mann–Whitney test, *n* = 7 mice per group). The thickness of the corpus callosum was measured from the same sections and sections at the levels of + 0.75 to −2.0 mm from Bregma. There was no statistically significant difference in males or females (Figure 7C). Since in L1/687 mice, the most prominent changes were observed in motor behavior, we first measured the site in the motor cortex, defined at the levels of + 1.70 to −1.30 mm from Bregma, according to Paxinos and Franklin [77]. There was no statistically significant difference either in males or females (Figure 7D).

**Figure 8:**
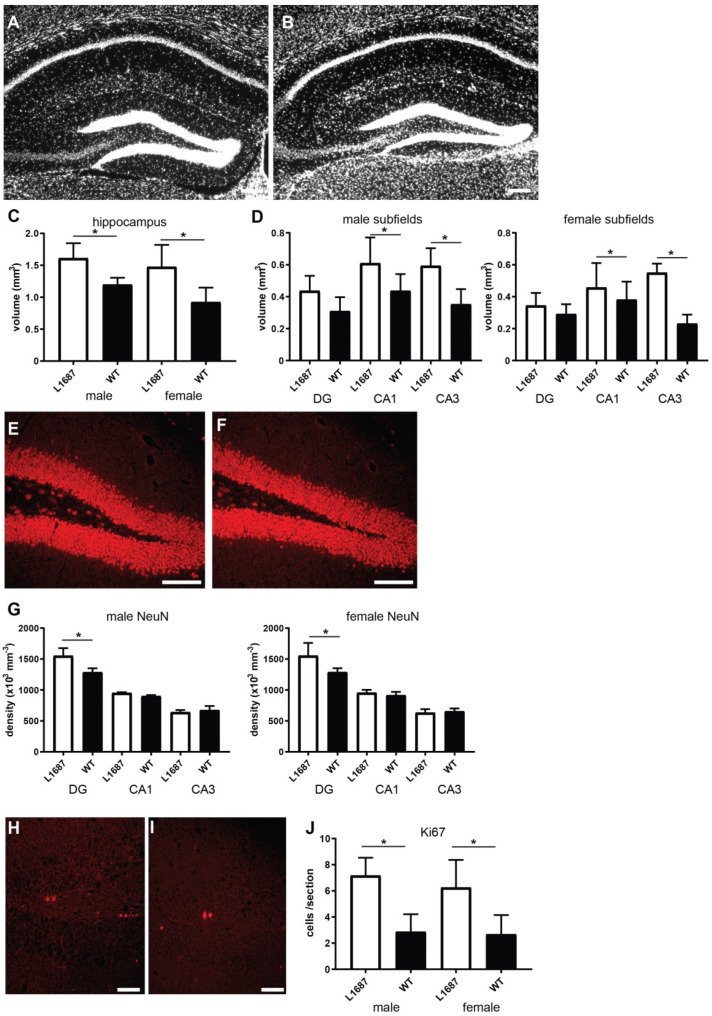
L1/687 mice show a larger hippocampus and more proliferating cells in the dentate gyrus (DG). (**A**,**B**) Shown is a DAPI stained coronal brain section from a female L1/687 (**A**) and a female WT (**B**) mouse. Scale bars: 200 µm. (**C**,**D**) Volume of the total hippocampus (**C**) and its subfields in male (left panel) and female (right panel) mice (**D**). Shown are mean values + SD; *t*-test, * *p* < 0.05; *n* = 3 per group for males and 4 mice per group for females. (**E**,**F**) NeuN immunostained hippocampus sections from a female L1/687 (**E**) and a female WT (**F**) mouse. Scale bars: 100 µm. (**G**) Shown are mean values + SD for densities (number of NeuN immunostained cells per mm^3^) in males (left panel) and females (right panel); *t*-test, * *p* < 0.05; *n* = 3 mice per group for males and 4 mice per group for females. (**H**,**I**) Ki67 immunostained DG sections from a female L1/687 (**H**) and a WT (**I**) mouse. Scale bars: 50 µm. (**J**) Shown are mean values + SD for the number of Ki67-positive cells in males and females; Mann−Whitney test, * *p* < 0.05; *n* = 3 mice per group for males and 4 mice per group for females.

We next investigated the hippocampus, a structure important in more elaborate cognitive behaviors. First, we measured the volume of the hippocampus according to Cavalieri’s principle, using DAPI-stained sections. The volume of the whole hippocampus as well as the volumes of the Cornu Ammonis-1 (CA1) and Cornu Ammonis-3 (CA3) subfields were higher in L1/687 males and females than in WT littermates (Figure 8A–D), while the volume of the DG tended to be larger in mutant animals. We also measured principal layer thickness in the CA1 and CA3 subfields and DG. A thicker granule cell layer was observed in the DG (56 ± 4.3 µm vs. 63 ± 5 µm for WT and L1/687 mice, respectively; *p* < 0.05, *t*-test, *n* = 7 mice per group), whereas there was no significant difference in the thickness of the pyramidal cell layers in CA1 (46.7 ± 3.3 µm vs. 52.2 ± 3.7 µm for WT and L1/687 mice, respectively; *p* > 0.05, *t*-test, *n* = 7 mice/genotype) and CA3 (125.3 ± 27.4 µm vs 130.2 ± 20.9 µm for WT and L1/687 mice, respectively; *p* > 0.05, *t*-test, *n* = 7 mice per group). The larger volume of the hippocampus could be due to larger numbers of neurons or glial cells, or a larger volume of dendrites and synapses. To clarify this, we counted the NeuN-positive neurons in the principal cell layers (granule cells in the DG and pyramidal cells in CA1 and CA3). Whereas the numbers of pyramidal neurons in the CA1 and CA3 were similar between genotypes, more granule cells were found in the DG of L1/687 mice than in WTs (Figure 8E–G and Supplementary Figure S10). Since DG granule cells are among the rare neurons which proliferate throughout life [78,79,80,81,82], we counted the number of Ki67-expressing proliferating cells in the subgranular layer. We discovered significantly more Ki67-positive cells in the DG of L1/687 mice than in WTs (Figure 8H–J).

We also counted the numbers of GFAP-positive astrocytes and radial glial cell precursors as well as Iba1-positive microglia but found no difference, neither in sex nor between genotypes (Supplementary Figure S11). We conclude that L1/687 mice have larger hippocampi than their WT littermates, more granule cells in the DG and higher proliferation of neural stem cells in the subgranular layer.

To conclude, L1/687 mice show sex-dependent changes in activity, social interest, anxiety and motor coordination. They also have a larger hippocampus and more granule cells in the DG, as well as more proliferating cells in the subgranular layer.

## 4. Discussion

In humans, mutations in L1 lead to the development of the L1 syndrome, which is characterized by intellectual disabilities, hydrocephalus of varying severity, spasticity and agenesis of the corpus callosum [41,42,67,68,83,84,85]. In addition, one case was described in which the whole L1 gene was deleted in a child with L1 syndrome [86]. Most of the nonsense and missense mutations found in the extracellular domain and the intracellular domain of L1 were shown to be disease-causing, while only few mutations were silent (http://www.l1cammutationdatabase.info/mutations.aspx; accessed 4 October 2023). However, the pathomechanisms underlying the neurodevelopmental defects caused by L1 mutations are not well understood. Of the almost 300 disease-causing L1 mutations found in humans, only few mutations have been investigated in more detail. L1 with mutations resulting in frameshift or nonsense changes was suggested to be eliminated from the cell surface or to be retained in the endoplasmic reticulum; e.g., investigation of L1 with R184Q mutation or D598N mutation in cultured cells showed that the mutated L1 proteins did not reach the cell surface [87]. Four mutations were shown in vitro to cause impaired cell-to-cell adhesion; although the mutated proteins reached the cell surface and were able to promote neurite outgrowth, one mutation resulted in impaired neurite outgrowth and neuronal migration, although the mutated protein reached the cell surface, and one mutated protein was shown to reach the cell surface and did not cause reduced cell adhesion [44,88,89,90]. In the cases where mutated L1 reached the cell surface and L1 functions were impaired, it was only seldom explored if homophilic L1-L1 interactions or heterophilic interactions of L1 with other proteins or L1 signaling were defective. Interestingly, of the mutations investigated in cell culture models, some of the human disease-causing mutations in the first FNIII domain did not interfere with L1 homophilic binding. L1 with mutation K655E was expressed at the cell surface, and homophilic binding was not affected, while L1 with the G698R mutation at the cell surface displayed reduced homophilic and heterophilic binding [91,92]. Of note, several mutations in the immunoglobulin-like and FNIII domains were suggested to affect the structural protein integrity without disrupting homophilic or heterophilic binding [91]. One animal model expressing mutant disease-causing L1 at the cell surface can be used to better understand and ameliorate L1 syndrome. Mice carrying the human disease-causing missense mutation p.D202N, which translates to D201N in mice, display the major pathological features of L1 syndrome, including behavioral impairments. The administration of agonistic L1 mimetics to cultures of primary neurons from L1-201 mice enhanced neurite outgrowth and neuronal migration and reduced neuronal cell death [44]. The R687A mutation investigated here leads to loss of the L1-70 fragment but unchanged expression of full-length L1 at the cell surface [53]. In addition, impaired mitochondrial transport and mitochondrial membrane potential in cultured neurons from L1/687 mice and normal mitochondrial complex I activity and lower adenosine triphosphate levels in the brain from L1/687 mice were found [54].

Since it is not well known which functions the fragments of L1 play in the nervous system in vivo and what the functional consequences are when cleavage sites are disrupted, we investigated whether the R687A mutation affects brain development and behavior of male and female mice. Normal muscle function and motor coordination of five-month-old L1/687 males and females were observed, while eight-month-old L1/687 males, but not L1/687 females, were impaired in motor coordination. Motor impairments were also observed in L1-deficient male mice in addition to weak and uncoordinated hind limbs [29,43,93]. A similar phenotype is observed in humans, where L1 mutations lead to limb spasticity [41,66,67,68]. These observations indicate the importance of L1 for motor functions and reveal that even point mutations in the extracellular domain of L1 affect L1’s functions in the nervous system. Furthermore, the defective proteolytic processing of L1 caused by deletion of the MBP cleavage site results in motor abnormalities in older males but not in females, suggesting that sex hormones also play a role in the development of motor deficits. That L1 can be regulated by hormones and interacts with hormone receptors was observed before. For instance, in the developing brain, L1 expression was regulated the by thyroid hormone [94], and in PC12 cells, L1 mRNA levels were enhanced after treatment with nerve growth factor [95]. Also, L1 interacts with estrogen and androgen receptors, topoisomerase 1, peroxisome proliferator-activated receptor and retinoid X receptors, and L1’s interactions with topoisomerase 1 and the peroxisome proliferator-activated receptor γ are mediated by L1-70 [27,29]. Thus, due to the different expression of hormones and hormone receptors in males and females, it is likely that the R687A mutation and deficiency in L1-70 lead to different behavioral changes in male and female mice. The morphological alterations and changes in motor coordination in L1/687 mice could be caused by impaired mitochondrial function, and we suggest that mitochondrial dysfunction can also contribute to the development of L1 syndrome. Since L1 could not be stimulated with function-triggering antibody 557 or L1 agonists in neurons from L1/687 mice [54], it is also likely that homophilic L1 interactions or L1 signaling are impaired in the R687A mutant. Since interactions of L1 with methyl CpG binding protein 2, estrogen receptor and retinoid X receptor were similar in wild-type and L1/687 neurons [27,33], we can assume that certain heterophilic interaction partners can interact with full-length L1 carrying the R/A687 mutation or with L1 fragments generated in L1/687 mice. In contrast, other heterophilic interactions depend on L1-70, like the interaction of L1 with microtubule-associated protein 1A/1B-light chain 3, and these are disturbed in L1/687 mice [55] and can contribute to the mutant phenotype. Our findings help to explain how mutations in L1 differently alter brain development and functioning, leading to moderate to severe L1 syndrome phenotypes, suggesting that enhancing mitochondrial and neuronal functions in L1 syndrome patients could help to ameliorate L1 syndrome. It is noteworthy to mention in this context, that the molecular mechanisms underlying impairments caused by most human L1 mutations are poorly understood and must be studied in the future to develop novel therapies for the treatment of L1 syndrome.

In addition to the alterations in motor coordination at 8 months of age, L1/687 males and females had a normal circadian rhythm in their home cage and were more active during the dark phase of the day but differed in their activity in a novel environment. L1/687 mice were more active in a novel environment, moved with a higher velocity and covered a larger distance, suggesting that the hyperactivity of L1/687 mice may be due to alterations in reaction to novelty. These mice also showed sex-specific differences in behavior. In contrast to L1/687 males, which have reduced anxiety-related behavior or reactions to novelty and show unchanged exploratory behavior, L1-deficient male mice displayed increased anxiety and impaired exploratory behavior [49]. The motor coordination of 8-month-old L1/687 males was reduced, while 8-month-old L1/687 females showed better coordination compared to the wild-type females, revealing gender differences for L1/687 mice. Furthermore, male and female mice with disruption of the dibasic sequences in the third FNIII homologous repeat of L1 (L1/858-863 mice), which also lack the L1-70 fragment [53], showed normal velocity and distance moved in the open field, and L1/858-863 females were less anxious [57]. These results support the notion that different hormone levels in males and females could underlie the differences in the behavior of L1/687 males and females. Furthermore, our results suggest that it is unlikely that the lack of L1-70 in mice is responsible for the enhanced activity of L1/687 animals in a novel environment, since L1/858-863 mice, also deficient in L1-70, did not show this abnormality.

Differences to L1-deficient mice were also observed regarding gender in social interactions. While male L1/687 mice visited the unfamiliar mouse more often, stayed closer and spent a longer time exploring to the unfamiliar mouse compared to WT males, L1/687 females spent similar times with familiar and unfamiliar mice. Impaired social interactions were also detected for L1-deficient male mice that were less interested in females in a social interaction paradigm [49]. Heterozygous L1 females were less interested in WT males [51]. Of note, in this context, is that L1 interacts with the methyl CpG binding protein 2 [33,73] and topoisomerase 1 [27], and that disruption of the L1-70 interaction with topoisomerase 1 leads to changes in the expression of the long autism genes neurexin 1 and neuroligin 1 [27]. Furthermore, L1 may play a role in autism spectrum disorders [74,75,96]. Based on these findings, we subjected L1/687 mice to the marble burying test to investigate if this mutation would lead to autism-like behavior or changes in reaction to novelty and anxiety. Yet, L1/687 males and females were normal. This distinguishes L1/687 mice from L1/858-863 mice, which also lack L1-70. L1/858-863 mice were socially normal, but L1/858-863 males buried more marbles than WT males [57]. In summary, the lack of L1-70 is not responsible for reduced social interest of L1/687 females nor does it lead to autism-like or compulsive-like behavior or changes in anxiety.

In an attempt to interpret the behavioral phenotypes on brain structural bases, we compared L1/687 mice to L1-deficient mice and mice expressing the L1 mutations C264Y or D201N. These mutants have enhanced ventricle size, abnormal pyramidal decussation and reduced thickness of the corpus callosum, and these changes can underlie impairments in cognition and behavior [43,44,46]. Interestingly, we did not find abnormalities in corpus callosum thickness or ventricle size in L1/687 mice but a larger hippocampus with a thicker granular layer and more proliferating granule cells in the DG. With the hippocampus being involved in cognition, learning, memory and mood [97], it is not astonishing that patients with major depression or posttraumatic stress disorder are reported to have a larger volume of ventricles, abnormal pyramidal decussation and reduced thickness of the corpus callosum [98,99]. Of note, the manipulation of DG circuits or DG neurogenesis in mice induces depression- and anxiety-like behaviors [100,101]. Importantly, the DG serves as a hippocampal input, as a preprocessor of incoming information and as a gate of sensory information to CA3 [102]. Aberrant adult neurogenesis in the DG has been reported to contribute to psychiatric and other neurological disorders [82]. Functional granule neurons are generated in the hippocampus throughout life via a multistep process that begins with GFAP-expressing radial glial cell precursors [103]. Astrocytes and radial neuronal precursor cells both express GFAP, the numbers of which were normal in the DG of L1/687 mice, suggesting that the generation of functional granule neurons is not affected in L1/687 mice. Hippocampal neural precursor cell proliferation is strongly increased by physical activity [104], and stress hormones reduce this adult neurogenesis [105]. The excitation/inhibition balance is of key importance for proper neurogenesis. In addition, excitation/inhibition imbalance may underlie aberrant functional integration of newborn neurons, which is associated with psychiatric and other neurological disorders [105]. Based on these studies and our finding that L1/687 males and females had increased locomotor activity and that L1/687 males were less anxious, we hypothesize that the higher activity of L1/687 mice contributes to increased neurogenesis in the DG and that L1/687 mice have reduced stress levels. The activation of prefrontal projections to the DG is important for social memory retrieval, and disruption or improper functioning leads to social memory deficits [106,107]. Since L1/687 females, but not L1/687 males, did not discriminate between known and unknown mice in social interactions, L1/687 females are likely to be deficient in regulating microcircuits that underlie social behaviors by affecting the input–output projections.

Here, we report that L1 mutant males with an R to A substitution at position 687 in the first FNIII domain differ from L1-deficient male mice and L1 syndrome model male mice. We propose that enhanced locomotion of L1/687 males and females and impaired social interactions of L1/687 females and, at more advanced ages, reduced motor coordination of L1/687 males could be caused by or lead to alterations in the DG of the hippocampus. Altogether, these findings confirm the view that mutations reveal their effects differently in males and females in an age-dependent manner. Although humans with L1/687 mutation have yet to be identified, our results encourage closer investigation of the known human L1 mutations for their behavior. Since only few L1 mutations where mutant protein is expressed at the cell surface were investigated with regard to their impact on brain morphology, behavioral features, L1 interactions and signaling, it is still impossible to clarify the exact mechanisms by which a mutation at a certain site in L1 affects L1 function and disease in individuals. Nonetheless, mutations in affected individuals might be remedied by modern gene technological methods, if FDA-approved, in the future, and even if only small abnormalities could be ameliorated, it is very likely that normalizing small differences would improve the quality of life.

## 5. Conclusions

L1/687 mice show enhanced locomotion, changes in social interactions and reduced motor coordination at older ages. Furthermore, L1/687 males and females have a larger hippocampus with more granule cells in the DG and a higher number of proliferating neural stem cells in the subgranular layer. Our findings indicate that mutating the MBP cleavage site in L1’s first FNIII domain impacts neurogenesis and the granule cell number in the DG and leads to sex- and age-dependent phenotypes, the occurrence of which remain to be understood in terms of molecular mechanisms.

## Data Availability

Data and materials will be made available upon reasonable request.

## Supplementary Materials

The following supporting information can be downloaded at: https://www.mdpi.com/article/10.3390/biom14040468/s1, Figure S1: Schematic presentation of L1 in mice expressing wild-type L1 or L1 with arginine to alanine exchange at position 687. Figure S2: Timeline of the behavioral experiments. Figure S3: Normal quadriceps muscle function of L1/687 mice. Figure S4: Normal motor performance of L1/687 mice in the pole test. Figure S5: Activity of L1/687 mice in the open field. Figure S6: Rearing, grooming and immobility time in the open field are similar between genotypes. Figure S7: Reduced immobility time of L1/687 males in the elevated plus maze. Figure S8: Normal social preference of L1/687 mice. Figure S9: Normal circadian rhythm of L1/687 mice. Figure S10: NeuN immunostained hippocampus sections from a male L1/687 (A) and a male WT (B) mouse. Figure S11: Normal numbers of GFAP-positive astrocytes and Iba1-positive microglia in the hippocampus of L1/687 mice.
