# Supplementary material for: Mice Mutated in the First Fibronectin Domain of Adhesion Molecule L1 Show Brain Malformations and Behavioral Abnormalities
Source: Biomolecules. 2024 Apr 11;14(4):468. doi: 10.3390/biom14040468 (PMC11048097; doi:10.3390/biom14040468)
Supplement: Supplementary file 1 [file biomolecules-14-00468-s001.zip › biomolecules-2889114-supplementary.pdf]

Supplementary Figures

Viviana Granato, Ludovica Congiu, Igor Jakovcevski, Ralf Kleene, Benjamin Schwindenhammer, Luciana Fernandes, Sandra Freitag, Melitta Schachner, Gabriele Loers: Mice mutated in the first fibronectin domain of adhesion molecule L1 show brain malformations and behavioral abnormalities.

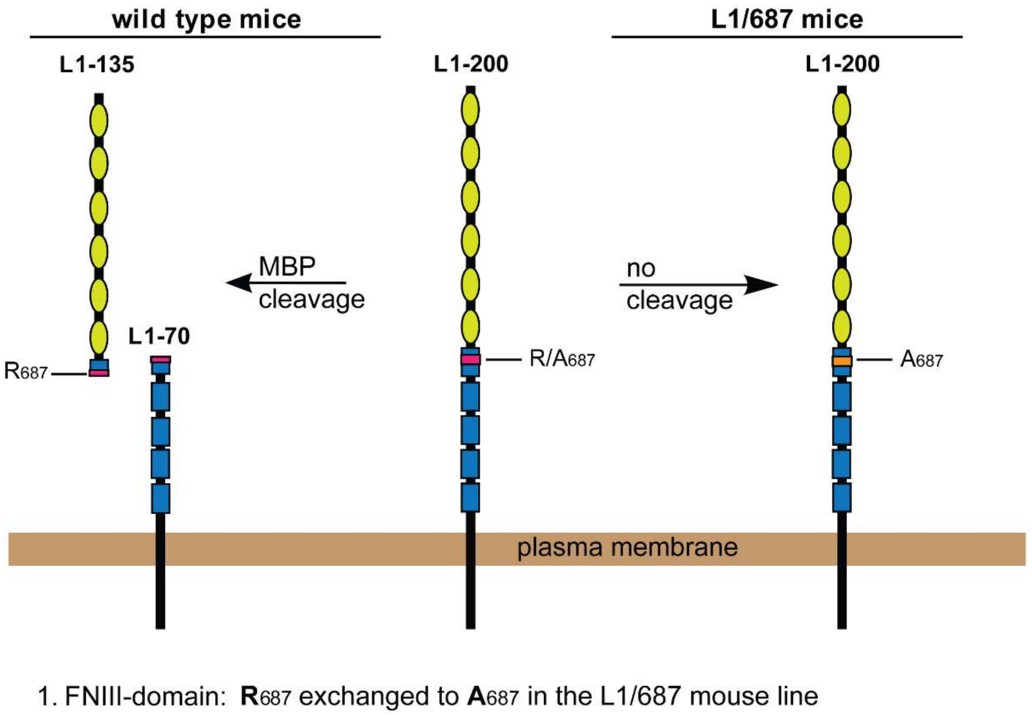

Supplementary Figure S1: Schematic presentation of L1 in mice expressing wild type L1 or L1 with arginine to alanine exchange at position 687. Full-length L1 (L1-200) consists of six immunoglobulin-like (green ovals) and five fibronectin type III homologous repeats (blue rectangles) in the extracellular domain, a transmembrane domain and an intracellular tail. Cleavage of L1 in its first FNIII domain at $R_{687}V$ by MBP generates 70 and 135 kDa fragments (L1-70 and L1-135, respectively) and exchange of R_{687} to A_{687} in the L1/687 mutant prevents the generation of these fragments.

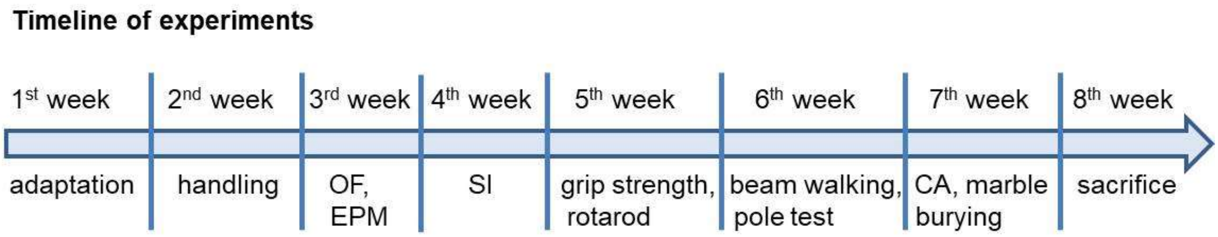

Supplementary Figure S2: Timeline of the behavioral experiments. Abbreviations: CA: circadian activity, EPM: elevated plus maze, OF: open field, PT: pole test, SI: social interaction.

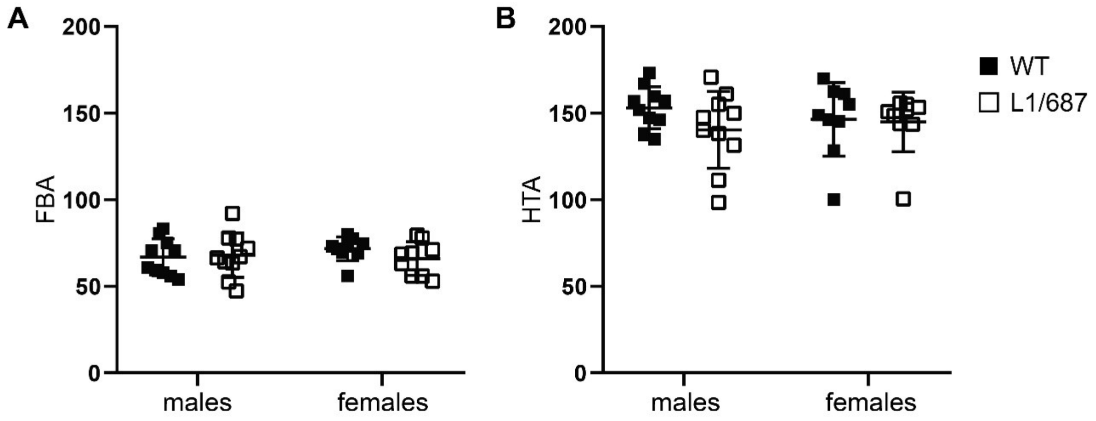

Supplementary Figure S3: Normal quadriceps muscle function of L1/687 mice. Four-month-old male and female WT and L1/687 mice were subjected to the beam walking test, and foot-base-angle (FBA; A) and heels-tail-angle (HTA; B) were determined. No impairments were seen for the L1/687 mutant mice. Single and average values ± SEM are shown; n = 9-10 mice per group; Mann–Whitney test; no significant differences were found, $p > 0.05$.

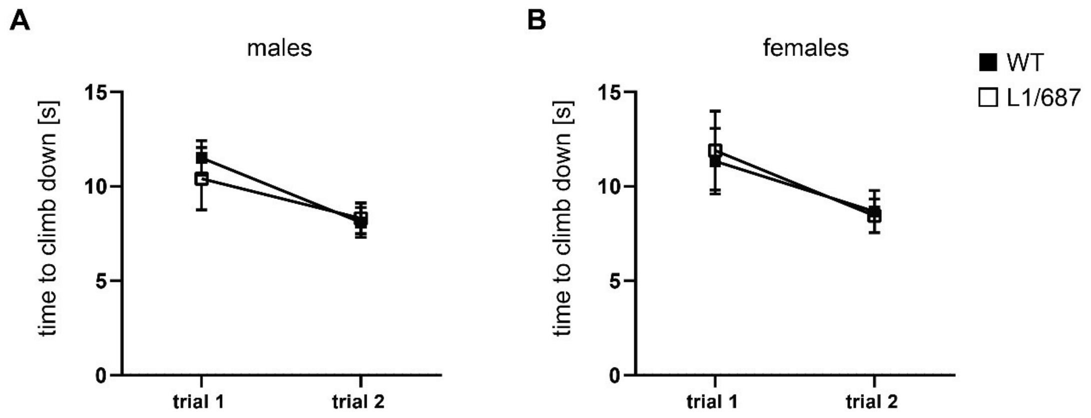

Supplementary Figure S4: Normal motor performance of L1/687 mice in the pole test. Four-month-old male (A) and female (B) WT and L1/687 mice were subjected to the pole test, and the time needed to climb down in two consecutive trials was measured. Average values ± SEM are shown; n = 10 males and 9 females per group; two-way ANOVA and Tukey´s multiple comparison post hoc test; no significant differences were found, $p > 0.05$.

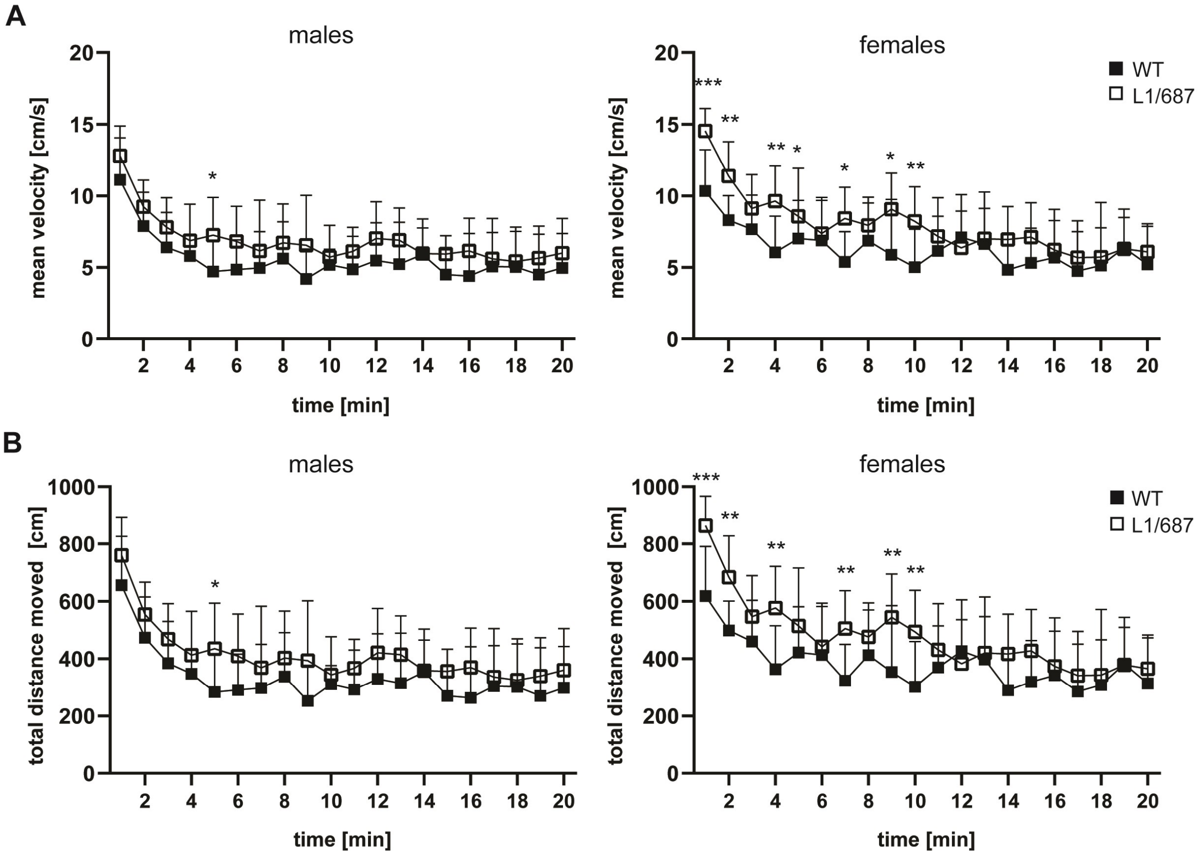

Supplementary Figure S5: Activity of L1/687 mice in the open field. Three-month-old male and female WT and L1/687 mice were subjected to the open field test, and the velocity (A) and distance moved (B) were determined over 20 min. Average values + SD are shown; n = 8-10 mice per group; three-way ANOVA and Bonferroni post hoc test, * $p < 0.05$, **$p < 0.01$, ***$p < 0.001$.

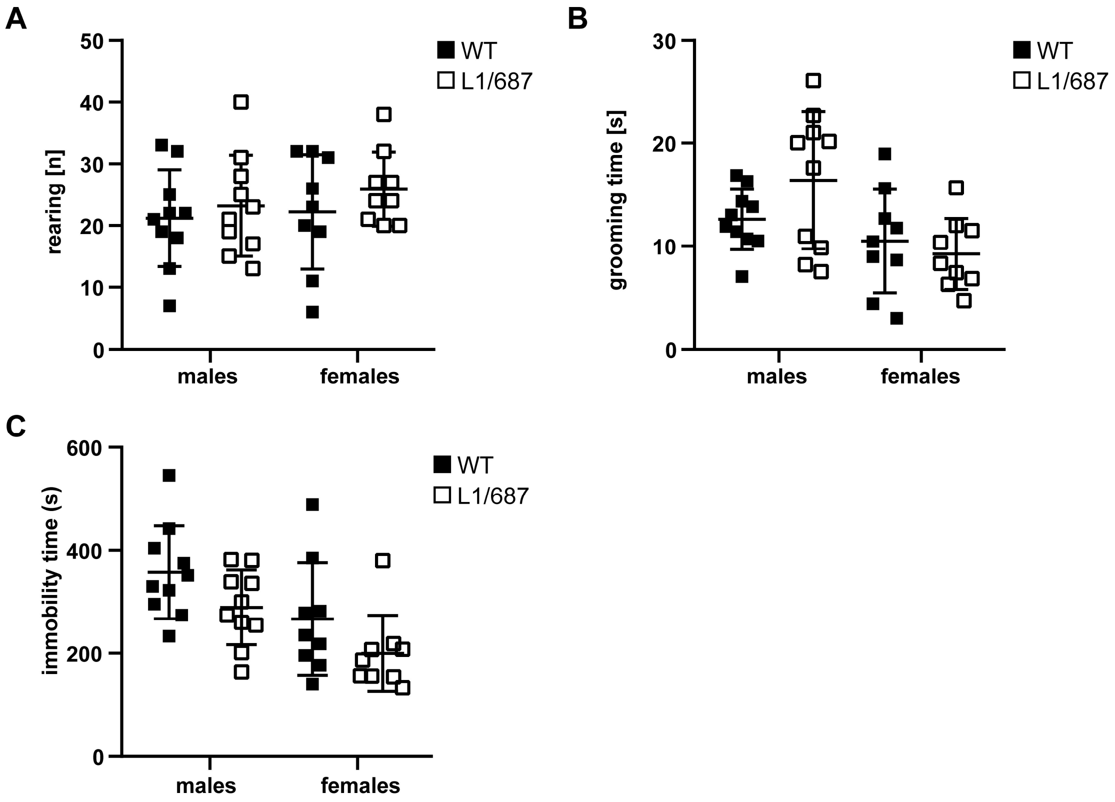

Supplementary Figure S6: Rearing, grooming and immobility time in the open field are similar between genotypes. Three-month-old male and female WT and L1/687 mice were subjected to the open field test, and the times that mice spent rearing (A), grooming (B) or were immobile were determined over 20 min. Single and average values ± SEM are shown; n = 9-10 mice per group; two-way ANOVA and Bonferroni post hoc test; no significant differences were found, $p > 0.05$.

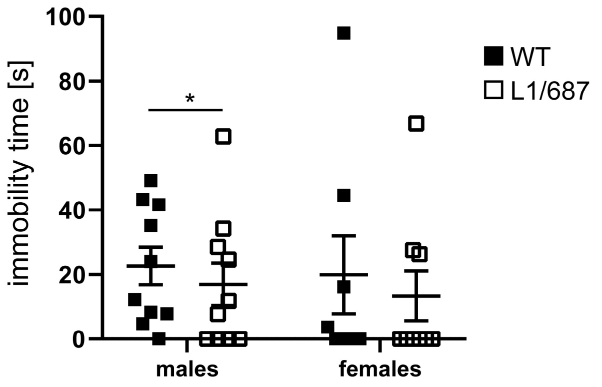

Supplementary Figure S7: Reduced immobility time of L1/687 males in the elevated plus maze. Three-month-old male and female WT and L1/687 mice were subjected to the elevated plus maze, and the times that mice were moving or immobile were determined. Single and average values ± SEM are shown; n = 9-10 mice per group; two-way ANOVA and Bonferroni post hoc test, * $p < 0.05$.

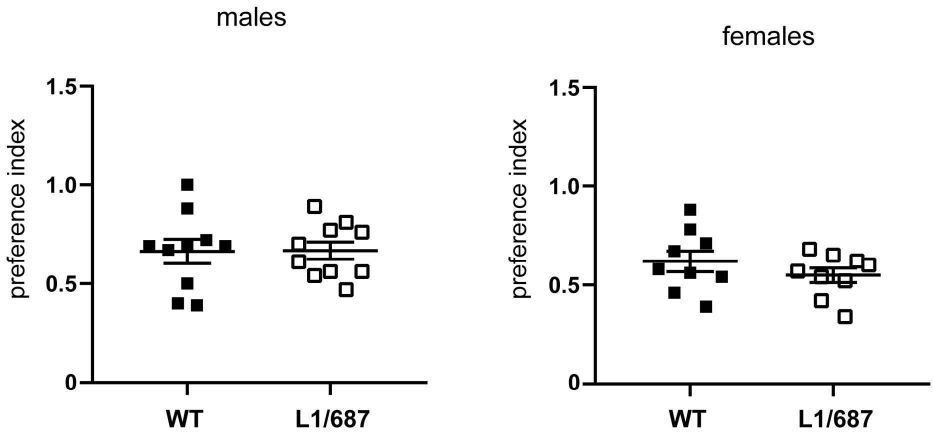

Supplementary Figure S8: Normal social preference of L1/687 mice. Four-month-old male and female WT and L1/687 mice were subjected to the social interaction test, and visits to the familiar and unfamiliar mice were recorded for 10 min. The preference index was then calculated: social preference index = time with unfamiliar mouse/(time with unfamiliar mouse + time with familiar mouse). For a genotype where time with unfamiliar mouse is significantly greater than time with familiar mouse, this represents the existence of social preference. Single and average values ± SEM

are shown; n = 9-10 mice per group; two-way ANOVA and Bonferroni post hoc test; no significant differences were found, $p > 0.05$.

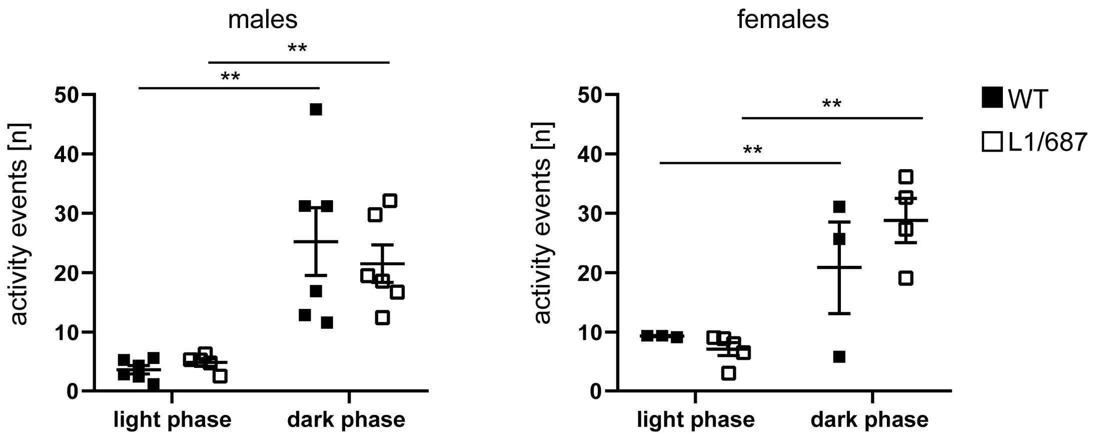

Supplementary Figure S9. Normal circadian rhythm of L1/687 mice. Home cage activity of five-month-old male and female WT and L1/687 mice was recorded for 24 h (7:00 am lights off, 7:00 pm lights on), and activity events were determined. Single and average values ± SEM are shown; n = 3-6 mice per group; two-way ANOVA and Bonferroni post hoc test, ** $p < 0.01$.

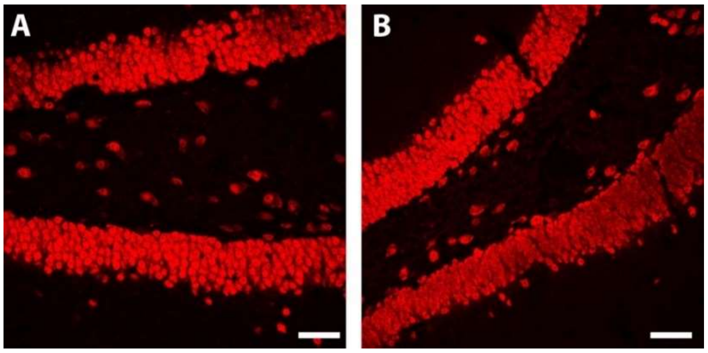

Supplementary Figure S10. NeuN immunostained hippocampus sections from a male L1/687 (A) and a male WT (B) mouse. Scale bars: 50 µm.

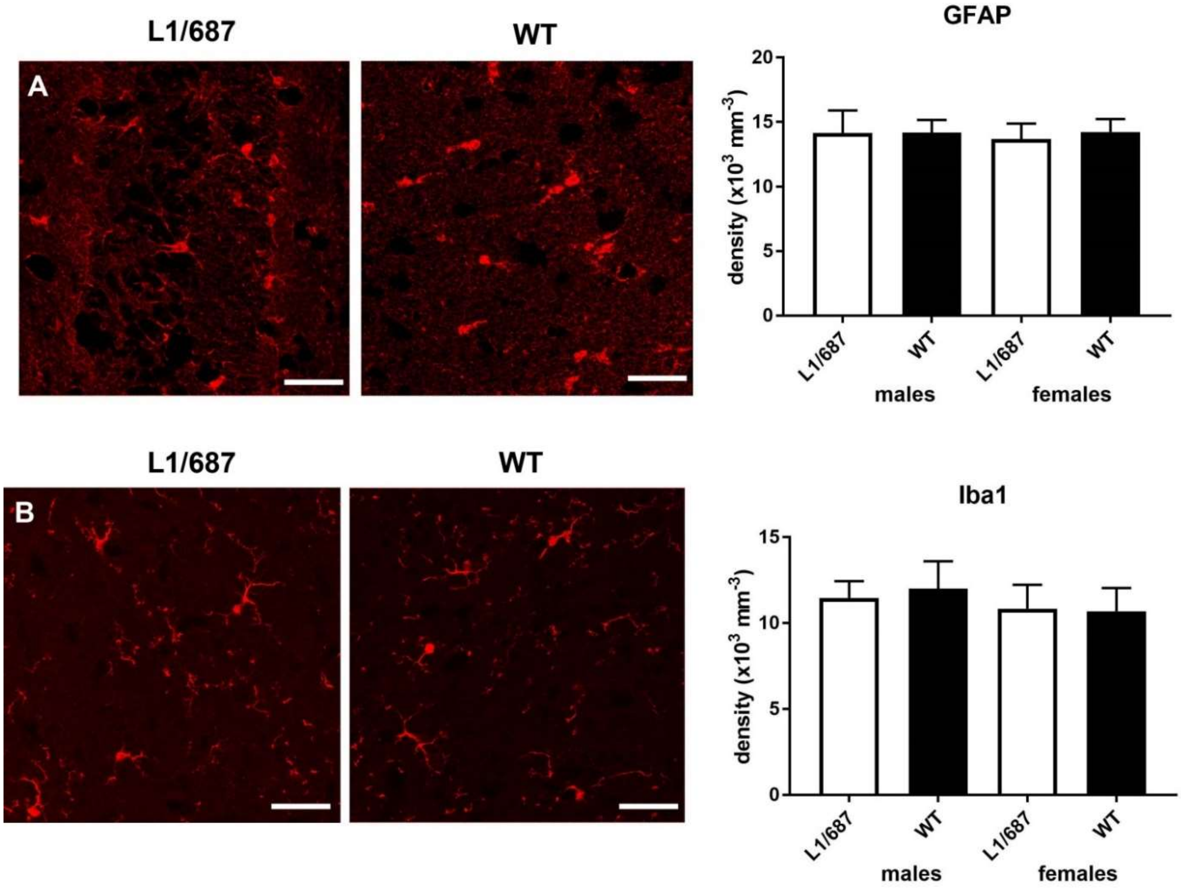

Supplementary Figure S11. Normal numbers of GFAP-positive astrocytes and Iba1-positive microglia in the hippocampus of L1/687 mice. (A) GFAP immunostained hippocampus sections from a female L1/687 and a female WT mouse. Scale bars: 50 µm. Graph: Mean values + SD for the density of GFAP-positive cells in males and females. (B) Iba1 immunostained hippocampus sections from a female L1/687 and a female WT mouse. Scale bars: 50 µm. Graph: Mean values + SD for the density of Iba1-positive cells in males and females. Mann−Whitney test, no significant differences, $p > 0.05$; $n = 3$ mice per group for males and 4 mice per group for females.
